# Triptolide alleviates hyperosmotic stress-induced human corneal epithelial cell damage by inhibiting NLRP3 inflammasome-mediated pyroptosis via the TLR4/NF-κB pathway

**DOI:** 10.3389/fmed.2026.1753857

**Published:** 2026-02-27

**Authors:** Ran Xia, Bei Zhan, Liming Tao

**Affiliations:** 1 Department of Ophthalmology, The Second Hospital Affiliated to Anhui Medical University, Hefei, China; 2 Department of Ophthalmology, Anhui No.2 Provincial People’s Hospital, Hefei, China

**Keywords:** corneal epithelial cells, NLRP3, pyroptosis, TLR4/NF-κB pathway, triptolide

## Abstract

**Purpose:**

To verify that Triptolide alleviates hyperosmotic stress-induced damage to human corneal epithelial cells (HCECs) by suppressing pyroptosis via the Toll-like receptor 4/nuclear factor-κB/NOD-like receptor family, pyrin domain-containing 3 (TLR4/NF-κB/NLRP3) axis.

**Methods:**

HCECs were divided into six groups: control (CG), hyperosmotic model (MG, 500 mOsm for 12 h), hyperosmotic model with LPS (MG + LPS), triptolide intervention (THSG, 30 nM in 500 mOsm medium), triptolide with LPS (THSG + LPS), and triptolide-only (TOG, 30 nM in standard medium). Cell viability was detected using Cell Counting Kit-8 (CCK-8). The pyroptosis rate was measured by flow cytometry, and lactate dehydrogenase (LDH) release was quantified to assess cytotoxicity. Inflammatory cytokines interleukin-1β (IL-1β) and interleukin-8 (IL-8) were measured by enzyme-linked immunosorbent assay (ELISA). The mRNA and protein expression levels of NLRP3 and NF-κB were analyzed by quantitative real-time polymerase chain reaction (qPCR) and Western blot, respectively. NF-κB p65 nuclear translocation was detected by immunofluorescence.

**Results:**

Compared to the CG (100.00 ± 0.00)%, MG significantly reduced HCECs proliferation to (54.47 ± 3.10)% and increased the pyroptosis rate to (40.28 ± 3.74)%. LPS further exacerbated these effects in the MG + LPS. The levels of inflammatory cytokines IL-1β and IL-8 were markedly elevated under hyperosmotic conditions, and further increased with LPS stimulation. THSG significantly ameliorated hyperosmotic-induced injury: cell proliferation increased to (86.47 ± 5.51)%, the pyroptosis rate decreased to (17.01 ± 2.33)%, and the release of IL-1β and IL-8 was substantially reduced. Triptolide also downregulated mRNA and protein expression of NLRP3 and NF-κB, and inhibited NF-κB p65 nuclear translocation. In the THSG + LPS group, triptolide partially reversed the LPS-enhanced inflammatory and pyroptotic responses, though its protective effect was attenuated compared to THSG without LPS. Notably, TOG showed no adverse effects on normal HCECs, with proliferation, pyroptosis rate, and inflammatory cytokine levels comparable to those of the CG.

**Conclusion:**

Triptolide alleviates hyperosmotic stress-induced pyroptosis and inflammatory injury in HCECs, likely through inhibiting the TLR4/NF-κB/NLRP3 pathway. This study suggests the potential of triptolide in treating hyperosmolarity-related ocular surface diseases.

## 1 Introduction

Tear hyperosmolarity is frequently associated with dry eye disease (DED), which negatively affects the function of human corneal epithelial cells (HCECs) and thereby contributes to corneal epithelial dysfunction, cell apoptosis, and the release of significant inflammatory factors. These effects significantly affect visual function and quality of life. As observed in the experimental model, a 500 mOsm hyper-osmotic medium impairs the proliferation and survival of HCECs, leading to increased levels of pro-inflammatory cytokines, including interleukin-1β (IL-1β) and tumor necrosis factor-α (TNF-α). This is associated with robust activation of mitogen-activated protein kinase (MAPK) signaling pathways, such as c-Jun N-terminal kinases (JNKs) and extracellular signal-regulated kinases (ERKs) (1–3). These investigations uncover the pivotal role of hyper-osmotic stress in ocular surface inflammation and signal an urgent need for a targeted intervention.

In the hyperosmolar-triggered inflammatory network, one of the essential upstream regulators is Toll-like receptor 4 (TLR4)/nuclear factor kappa B (NF-κB) signaling. TLR4 recognizes endogenous danger signals and induces NF-κB, which then triggers the transcriptional up-regulation of pro-inflammatory genes (e.g., IL-1, TNF-α) (4). Furthermore, intracellular trafficking of the TLR4/myeloid differentiation factor 2 (MD-2) complex directly contributes to lipopolysaccharide-mediated inflammatory responses (5). In addition, the NOD-like receptor family, pyrin domain-containing 3 (NLRP3), inflammasome is activated in a hyper-osmotic environment. Reactive oxygen species-dependent NLRP3 activation promotes IL-1β maturation and secretion to a greater degree, thereby creating a positive feedback loop between TLR4/NF-κB and NLRP3 (6, 7). It has been reported that JNK inhibitors, glucocorticoids, and antioxidants can mitigate this pathway; however, there are also limitations to directly targeting this damaging pathway, such as undesirable drug effects, limited target exclusivity, and ineffective protection of corneal epithelial cells. For instance, JNK inhibitors can inhibit matrix metalloproteinase 9 (MMP-9) expression, but they cannot block NLRP3 inflammasome activation (3). Thus, traditional glucocorticoids block NF-κB but may also increase intraocular pressure and cause cataracts (8). Thus, it is necessary to develop a safe and efficient agent that simultaneously inhibits TLR4/NF-κB signaling and the NLRP3 inflammasome.

Hyperosmotic stress, primarily resulting from tear film instability and increased evaporation, is a hallmark feature of DED (9). It triggers a cascade of pathogenic events on the ocular surface, including the activation of stress-responsive signaling pathways such as MAPK, the upregulation of pro-inflammatory cytokines, and the induction of oxidative stress (10). These processes collectively contribute to corneal epithelial barrier dysfunction, goblet cell loss, and nerve sensitization, ultimately leading to clinical symptoms of discomfort, visual disturbance, and chronic inflammation. Moreover, sustained hyperosmolarity perpetuates a vicious cycle by further promoting inflammatory mediator release and compromising tear film homeostasis, thereby exacerbating DED progression. Understanding the multifaceted role of hyperosmotic stress in ocular surface pathophysiology is crucial for developing targeted therapeutic strategies to interrupt this detrimental cascade.

Based on the restricted therapies for hyper-osmotic stress-induced corneal epithelial cell injury, this work provides a multipath intervention strategy when using triptolide. Triptolide can reduce TLR4 expression and down-modulate lipopolysaccharide-induced NF-κB phosphorylation, thereby markedly decreasing cytokines such as TNF and IL-1β in models of acute lung inflammation (11). Triptolide blocks NF-κB activation in immune cells by inducing the expression of inhibitors of kappa B alpha (IκBα), thereby potently suppressing NF-κB signaling (12). According to these results, the present study systematically explored and analyzed the possible mechanism by which triptolide regulates the NLRP3 inflammasome via the TLR4/NF-κB signaling pathway in hyperosmolar-cultured HCECs. The goal of the combination is to simultaneously inhibit two major inflammatory pathways, representing a more complete, molecularly tailored approach for dry eye and ocular surface diseases.

## 2 Materials and methods

### 2.1 Experimental materials and instruments

HCECs were obtained from Shanghai Lianmai Bioengineering Co., Ltd. and cultured in DMEM/F12 medium (Servicebio, G4515-500ML) containing 10% FBS and 1% penicillin/streptomycin. All cells were kept routinely in a humidified incubator at 37 °C with 5% CO_2_. The primary reagents used in this study were: Cell Counting Kit-8 (CCK-8) kit (Servicebio, G4103-5ML), RNA Easy Fast Animal Tissue/Cell Total RNA Extraction Kit (TIANGEN, DP451-TA), Crystal Violet Staining Solution (Servicebio, G1101-500ML), BeyoRT II cDNA First Strand Synthesis Kit (Beyotime, D7168M), BeyoFast SYBR Green qPCR Mix (Beyotime, D7262-25 mL). Main instruments used: mini centrifuge (Servicebio, D1008E), decolorizing shaker (Servicebio, TSY-B), pipettes (Dragon, KE0003087/KA0056573), upright fluorescence microscope (Nikon Eclipse C1, Japan), gel imaging system (Tanon, CRB F119), real-time PCR instrument (BIO-RAD, CHROMO4).

### 2.2 Experimental methods

#### 2.2.1 Cell culture and experimental grouping

The HCECs were removed from liquid nitrogen and quickly thawed in a 37 °C water bath with mild agitation. The cell suspension is then added to a 15 mL centrifuge tube in a sterile manner. Then, 1 mL of complete medium (DMEM/F12 supplemented with 10% FBS and 1% penicillin/streptomycin) was added, and the mixture was mixed well. The tube was then centrifuged at 1,000 rpm and room temperature for 5 min. The cell pellet was re-suspended in 1 mL of complete medium and plated onto culture dishes for subsequent incubation at 37 °C with 5% CO_2_.

The cells were passaged at 80–90% confluence. In brief, the old medium was removed, and the cells were washed twice with PBS. After being washed twice with PBS, 1 mL of 0.25% Ethylenediaminetetraacetic acid (EDTA)-trypsin was added to cover the cell layer, and the culture vessel was returned to the incubator for about 5 min. When cells began to round and dissociate under microscopic observation, 1 mL of complete medium was added to inactivate the trypsin. The supernatant was carefully pipetted to form a single-cell suspension, and the cells were collected into a 15 mL centrifuge tube following centrifugation at 1,000 rpm for 5 min. The supernatant was removed, and the pellet was resuspended in 2 mL of complete medium. The cell suspension was then equally divided into two fresh culture plates.

For plated experiments, PBS, trypsin, and complete medium were equilibrated in a water bath before use. Cells were plated at a density of 8 × 104 cells per well in 6-well plates, 4 × 104 cells per well in 12-well plates, 2 × 104 cells per well in 24-well plates, and 5,000 cells per well in 96-well plates culture dishes after digestion and centrifugation. The plates were then incubated at 37 °C and 5% CO_2_ for 18–24 h before further experimental procedures.

The cells were divided into the following six experimental groups: control group (CG), in which cells were cultured under standard conditions without any treatment; model group (MG), in which cells were treated with 500 mOsm hyperosmotic medium to establish a damage model; Lipopolysaccharide (LPS)-treated hyperosmotic-stressed cell group (MG + LPS), in which cells were treated with 500 mOsm hyperosmotic medium to establish the damage model and then further stimulated with LPS; triptolide-treated hyperosmotic-stressed cell group (THSG), in which cells were first exposed to hyperosmotic medium and subsequently treated with 30 nM triptolide; LPS and triptolide co-treated hyperosmotic-stressed cell group (THSG + LPS), in which cells were first stimulated with hyperosmotic medium and then co-treated with 30 nM triptolide and LPS; and triptolide-only group (TOG), in which cells were treated solely with 30 nM triptolide under standard culture conditions. The stimulating concentration of LPS is 100 ng/mL. According to the literature (13), triptolide at 1–30 nM has no cytotoxicity on HCECs but effectively inhibits the NF-κB pathway. Thereafter, 30 nM Triptolide was employed in the present study.

#### 2.2.2 Cytotoxicity assay and hyperosmotic model establishment

The 500 mOsm hyper-osmotic medium was prepared by adding a filtered, sterilized stock solution to isotonic culture medium (DMEM/F12 + 10% FBS, ~312 mOsm). Approximately 90 μL of 1 M NaCl (final concentration, 90 mM NaCl) was required for a final osmolarity of 500 mOsm per milliliter of medium. The solution was well stirred, and the final osmolarity was confirmed to be 500 ± 10 mOsm using an osmometer.

HCECs were seeded at an appropriate density in 96-well plates. After 24 h of incubation to allow for cell adhesion, the culture medium was aspirated and replaced with the prepared 500 mOsm hyper-osmotic medium. Cells were then exposed to this hyper-osmotic stress for varying durations (2, 6, 12, 18, and 24 h), with a group maintained in isotonic medium serving as the blank control. Cell proliferation was evaluated using the CCK-8 kit according to the manufacturer’s instructions. In short, 10 μL of CCK-8 solution was added to each well and incubated at 37 °C for two hours. A microplate reader measures absorbance at 450 nm. Cell viability was analyzed in triplicate for each condition, and the average values were calculated. Based on cytotoxicity findings, a 12-h treatment with 500 mOsm hyper-osmotic medium, resulting in a survival rate of about 50%, was adopted as the standard condition for inducing hyper-osmotic injury in all experiments. This model is also consistent with previous reports of enhanced apoptosis induced by 90 mM NaCl in the culture medium (500 mOsm) (14).

#### 2.2.3 Cell morphology observation

After 12 h of treatment with the appropriate experimental conditions, HCECs were observed and photographed using an inverted microscope. Morphological changes, including cell shrinkage and changes in cellular morphology, were noted and photographed at 200× magnification.

#### 2.2.4 Flow cytometry for pyroptosis detection

Following 12 h of treatment, HCECs were harvested for pyroptosis analysis. The cell culture supernatant was collected and retained. The adherent cells were washed with cold PBS three times, then detached by adding 500 μL of 0.25% (w/v) EDTA-free trypsin per well. The reaction was stopped by adding a complete medium to 1 mL. The floating cells (top layer) were mixed with the supernatant, gently pipetted to form a single-cell suspension, and then transferred to a centrifuge tube. The tube was centrifuged at 1,500 rpm for 5 min at 4 °C. After removing the supernatant, the cell pellet was washed twice with cold PBS (centrifugation conditions: 1,500 rpm for 5 min at 4 °C). The pellet was resuspended in 500 L of 1× binding buffer. Then, 5 μL of Annexin V-FITC and 2 μL of propidium iodide (PI) were added to the cell suspension, each in turn, and the mixture was gently mixed. After incubation for 10 min in the dark at room temperature, the samples were analyzed by flow cytometry. The excitation and emission wavelengths were 488 nm and 530 nm, respectively. The ratio of pyroptosis cells was analyzed using FlowJo software (Version 10.7).

#### 2.2.5 ELISA for inflammatory factors

After 12 h of treatment, the levels of inflammatory factors in the culture supernatant of HCECs were determined by using ELISA kits. The reagents and samples were prepared and brought to room temperature before the assay. The presence of burgeoning standards or diluted samples in the appropriate wells was warranted. Fifty microliters of the supplied detection antibody cocktail was then added to each well. The plate was sealed and incubated for 1 h at room temperature on a plate shaker, adjusted to 400 rpm. After incubation, the plates were washed three times with 350 μL of 1× Wash Buffer. For each wash, the buffer was allowed to remain in the wells for at least 10 s before suctioning. After the last wash, the plate was inverted and thoroughly blotted onto clean absorbent paper to ensure no liquid remained. The 3,3,5,5-Tetramethylbenzidine (TMB) substrate solution was added to each well, and the plate was placed on the plate shaker (400 rpm) in the dark for 10 min. The enzymatic reaction was terminated by adding 100 μL Stop Solution to each well, then shaking the plate for 1 min to mix thoroughly. The optical density at 450 nm was measured immediately with a microplate reader. The absorbance’s of the standard concentrations were plotted to create a standard curve, from which amounts of IL-8 and IL-1β in the sample could be interpolated.

#### 2.2.6 Immunofluorescence staining for NF-κB p65 nuclear translocation

Logarithmically growing HCECs were enzymatically digested with trypsin and seeded onto aseptic cover slips in 24-well plates. Following 24 h of incubation at 37 °C in a humidified atmosphere of 5% CO_2_, cells were treated as appropriate. The coverslips were then carefully removed from the dishes after treatment. The coverslip cells were fixed with 4% paraformaldehyde for 30 min at room temperature and washed three times (5 min each) with PBS. Permeabilisation was performed with 0.3% Triton X-100 for 10 min, followed by blocking of non-specific binding sites with 2% bovine serum albumin (BSA) for 30 min. Thereafter, the cells were incubated overnight at 4 °C in a humidified chamber with the NF-κB p65 primary antibody (1:100 diluted in blocking buffer). The following day, the free primary antibody was washed out by rinsing the coverslips three times with PBS. The cells were incubated in the dark with a species-specific fluorescently labeled secondary antibody for 1 h. The coverslips were removed from the wells and washed 3 times with PBS (gently) to remove residual secondary antibody. The paper filter was used to blot any remaining PBS from the surface lightly. Finally, the coverslips were mounted onto glass slides with anti-fade mounting medium and stained for nuclei with DAPI. The slides were left to dry for a minimum of 10 min before imaging. The fluorescence signal was detected and recorded using a fluorescence microscope. The fluorescence intensity, which reflects nuclear translocation of NF-κBp65, was analyzed using ImageJ.

#### 2.2.7 Quantitative real-time PCR for gene expression analysis

After 12 h of treatment, total RNA from HCECs was harvested according to the manufacturer’s instructions using the RNeasy Fast Animal Tissue/Cell Total RNA Extraction Kit. The concentration and purity (A260/A280 ratio) of the RNA were determined by spectrophotometry. Total RNA was reverse-transcribed into complementary DNA (cDNA) using the BeyoRT II cDNA First Strand Synthesis Kit, according to the manufacturer’s instructions. qPCR was conducted with BeyoFast SYBR Green qPCR Mix in a 20 μL reaction made up of 10 μL TB Green Premix Ex Taq II, 1 μL forward primer (10 μM), 1 μL reverse primer (10 μM), 2 μL cDNA template, and 6 μL nuclease-free water. The thermal cycling conditions were: pre-denaturation at 95 °C for 30s; 40 cycles of PCR reaction (95 °C for 5 s, 57.5 °C for 34 s); and a melting curve stage (95 °C for 15 s, 60 °C for 60s, 95 °C for 15 s). The 2^−ΔΔCt^ method was used to calculate the relative RNA expression levels, with glyceraldehyde-3-phosphate dehydrogenase (GAPDH) as the internal reference gene. The mRNA expression changes of NLRP3 and NF-κB were detected. The sequences of PCR primers are detailed in Table 1.

**Table 1:** Primer sequences for quantitative real-time PCR.

Gene name	Primer direction	Primer sequence (5′ → 3′)
NLRP3	Forward primer	5′-AGGAGGAAGAAGAAGAGAGGA-3′
	Reverse primer	5′-AGAGACCACGGCAGAAGC-3′
NF-κB	Forward primer	5′-ATGGCTTCTATGAGGCTGAACTCTG-3′
	Reverse primer	5′-TTGCTCCAGGTCTCGCTTCTTC-3′
GAPDH	Forward primer	5′-TGGCCTTCCGTGTTCCTAC -3′
	Reverse primer	5′-GAGTTGCTGTTGAAGTCGCA −3′

#### 2.2.8 Western blot analysis for protein expression

HCECs were lysed for Western blotting at 12 h post-treatment. Cells were washed twice with ice-cold PBS and lysed in radio immunoprecipitation assay (RIPA) buffer containing protease and phosphatase inhibitors on ice for 30 min. The cell lysates were spun for 30 min at 13,000 rpm (4 °C) to remove debris. The supernate, as the total protein extract, was recovered. Protein levels were quantified using the BCA assay to ensure equal loading. Protein samples were loaded and resolved by electrophoresis on a 12% sodium dodecyl sulfate-polyacrylamide gel (SDS-PAGE) and electro blotted onto polyvinylidene fluoride (PVDF) membranes. The membrane was blocked for 1 h at room temperature with 5% (w/v) non-fat milk in Tris-buffered saline containing Tween 20 (TBST) to block non-specific binding. After blocking, the membrane was washed three times with TBST (5 min each) and then incubated overnight at 4 °C with primary antibodies against NLRP3, NF-κB, and Gasdermin D N-terminal fragment (GSDMD-N), at a dilution of 1:1,000. After incubation with the primary antibodies, the membrane was washed with TBST, then probed with HRP-conjugated secondary antibodies (diluted 1:1,000) for 1 h at room temperature. After being washed three times with TBST, protein bands were detected using an enhanced chemiluminescence (ECL) detection kit, visualized according to the manufacturer’s protocol, and imaged on a Tanon CRB F119 image system. GAPDH served as an internal loading control to calculate the relative expression of the target proteins NLRP3, NF-κB, and GSDMD-N. All experiments were carried out with at least 3 independent biological replicates.

#### 2.2.9 Detection of lactate dehydrogenase

In this experiment, the Lactate Dehydrogenase (LDH) Cytotoxicity Assay Kit (Beyotime, C0016) was used to measure LDH activity. After collection, cells were centrifuged, and the supernatant was discarded. The cell pellet was disrupted by ultrasonic lysis, followed by centrifugation at 8,000*g* for 10 min at 4 °C. The resulting supernatant was placed on ice for subsequent analysis. Absorbance was measured at 450 nm using a microplate reader (SpectraMax iD3). A standard curve was prepared from serially diluted standards (0–20 μmol/L). LDH activity in the samples was calculated based on the standard curve according to the formula: L-LDH (U/mL) = 66.67 × A × F, where “A” is the sample concentration (μmol/mL) and “F” represents the dilution factor. One unit of enzyme activity was defined as the amount of LDH required to catalyze the formation of 1 nmol of pyruvate per minute per milliliter of sample.

### 2.3 Statistical analysis

All experiments were carried out with at least 3 independent biological replicates. Values are presented as the mean ± standard deviation (SD). Data were analyzed with SPSS software (Version 26.0). Normality of the data distribution was examined with the Shapiro–Wilk test. To compare more than two groups, one-way analysis of variance (ANOVA) was used for data that conformed to the normality and homogeneity assumptions. When the ANOVA test showed *p* < 0.05, the Least Significant Difference (LSD) *post hoc* test was performed to compare these groups. *p* < 0.05 was considered statistically significant in all tests.

## 3 Results

### 3.1 Cell morphology

**Figure 1:**
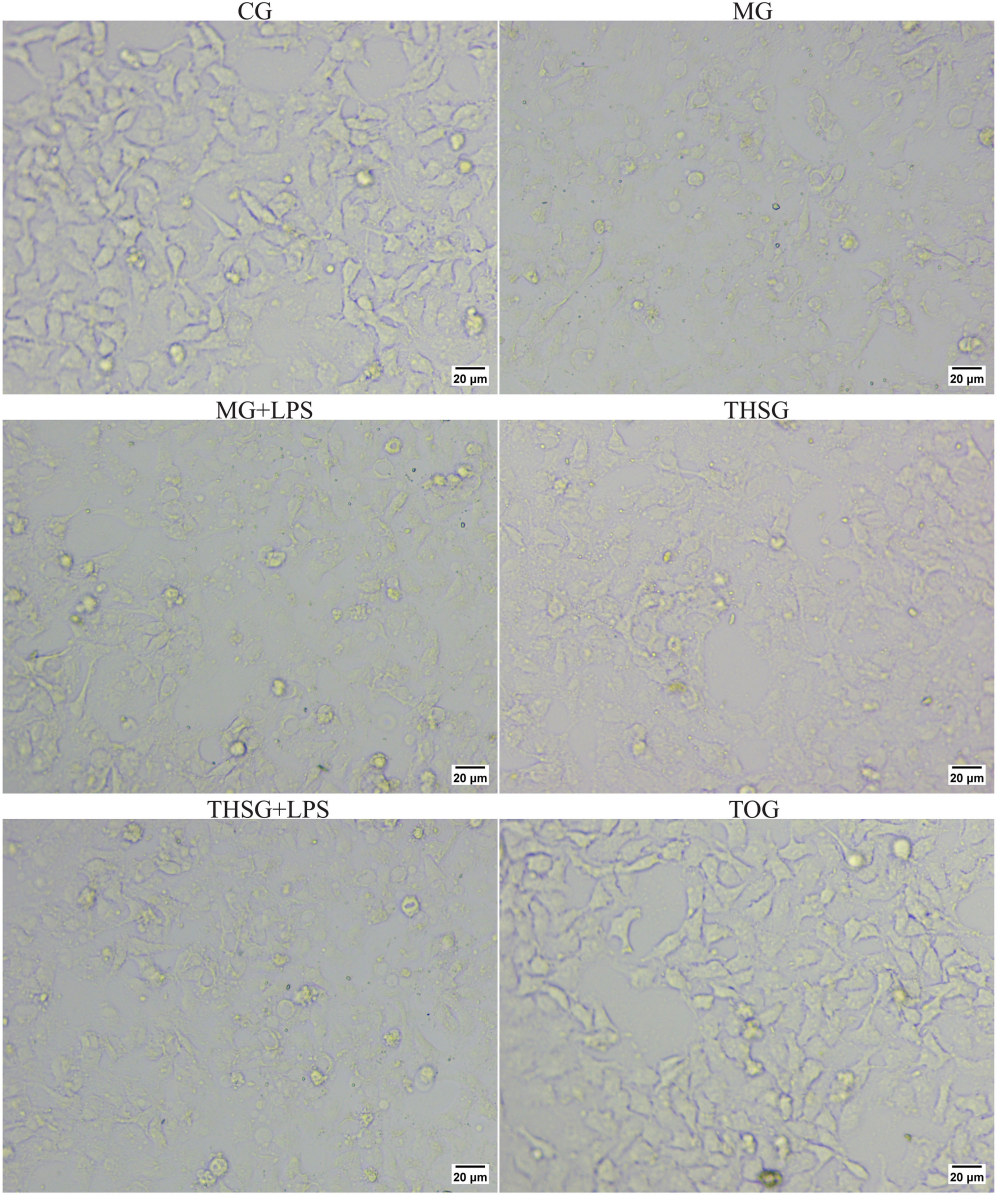
Morphological changes of human corneal epithelial cells (HCECs) under different treatment groups. HCECs were observed and photographed under an inverted microscope at 200× magnification after 12 h of culture under respective conditions. Representative images are shown, illustrating the regular morphology in the Control Group (CG); the significant pathological changes such as cell shrinkage and rounding induced by hyperosmotic stress (MG) and hyperosmotic stress plus LPS (MG + LPS); the amelioration of these hyperosmosis-induced changes by triptolide intervention (THSG); the partial attenuation of this protective effect when triptolide was co-administered with LPS under hyperosmotic stress (THSG + LPS); and the similar morphology to CG in the triptolide-only group (TOG).

The CG cell morphology was regular. Compared with the CG, a large number of cells in the MG, MG + LPS, and THSG groups showed contraction, with cell shapes changing from irregular rhomboids to round. Compared with the MG, MG + LPS, and THSG + LPS groups, the degree of contraction was reduced in the THSG group, with only a few cells exhibiting severe contraction. Notably, the cell morphology in the TOG group was similar to that of the CG group. Details are shown in Figure 1.

### 3.2 Proliferation rate of HCECs

**Figure 2:**
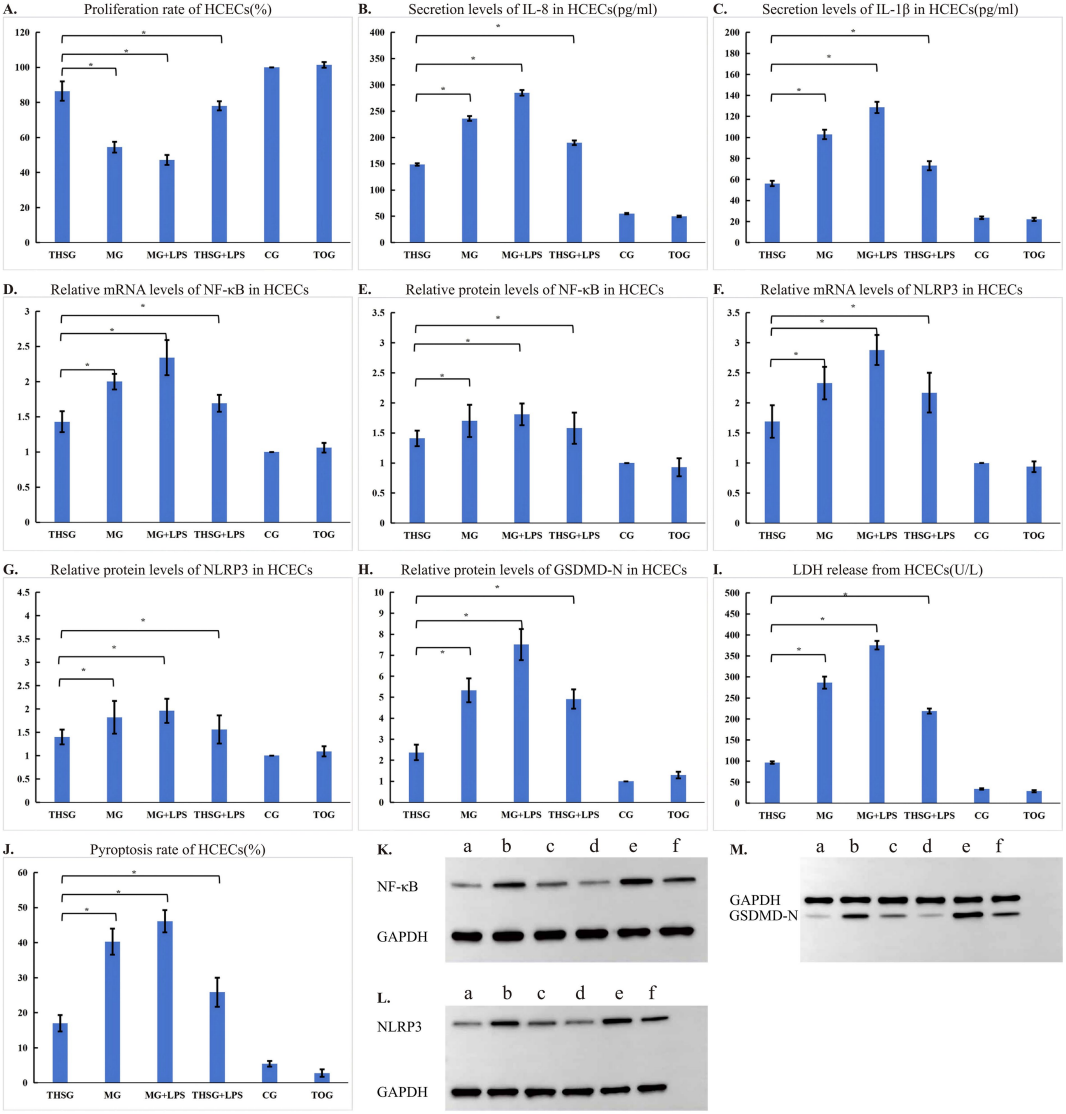
Effects of hyperosmotic stress, LPS, and triptolide on proliferation, inflammatory response, NF-κB/NLRP3 signaling, and pyroptosis in HCECs. **(A)** Cell proliferation rate (CCK-8 assay). **(B,C)** Levels of inflammatory cytokines IL-1β and IL-8 in the culture supernatant (ELISA). **(D,F)** Relative mRNA expression levels of NLRP3 and NF-κB (qPCR analysis). **(E,G,H,K–M)** Relative protein expression levels of NLRP3, NF-κB, and the pyroptosis execution protein GSDMD-N (Western blot analysis, bands show representative images). **(I)** Lactate dehydrogenase (LDH) is released in the culture supernatant. **(J)** Cellular pyroptosis rate detected by flow cytometry (Annexin V-FITC/PI double staining). **(A)** Control group, CG; **(B)** Model group, treated with hyperosmotic stress, MG; **(C)** Hyperosmotic stress plus LPS, MG + LPS; **(D)** Triptolide intervention, THSG; **(E)** Triptolide was co-administered with LPS under hyperosmotic stress, THSG + LPS; **(F)** Triptolide-only group, TOG. All quantitative data are presented as mean ± SD (*n* = 3 independent experiments). Statistical significance between groups was determined by one-way ANOVA followed by the LSD post-hoc test. *p < 0.05.

The proliferation rates of HCECs differed significantly among the groups (*F* = 4,004.35, *p* < 0.001). The proliferation rate in the CG was (100.00 ± 0.00)%. In comparison, the MG was more sensitive to hyperosmotic stress, and its proliferation rate (54.47 ± 3.10%) was significantly lower than that in the CG (*p* < 0.05). The proliferation rate in the MG + LPS was further reduced to (47.17 ± 2.90)%, which was also significantly lower than that in the MG (*p* < 0.05). The proliferation rate in the THSG was (86.47 ± 5.51)%, which was significantly higher than that in the MG and MG + LPS (both *p* < 0.05) but remained lower than that in the CG (*p* < 0.05). The proliferation rate in the THSG + LPS was (78.02 ± 2.58)%, significantly lower than that in the THSG (*p* < 0.05) but significantly higher than those in the MG and MG + LPS groups (both *p* < 0.05). The proliferation rate in the TOG was (101.42 ± 1.61)%, showing no significant difference from the CG but was significantly higher than those in the MG, MG + LPS, THSG, and THSG + LPS (all *p* < 0.05). The results are presented in Table 2 and Figure 2A.

**Table 2:** Proliferation rate of HCECs (%).

Groups	Proliferation rate of HCECs (%)	*F*	*p*
CG	100.00 ± 0.00	4,004.35	<0.001
MG	54.47 ± 3.10^bcd^		
MG + LPS	47.17 ± 2.90^acd^		
THSG	86.47 ± 5.51^abd^		
THSG + LPS	78.02 ± 2.58^abc^		
TOG	101.42 ± 1.61		

^a^P, compared with the MG (*p* < 0.05); ^b^P, compared with the MG + LPS (*p* < 0.05); ^c^P, compared with the THSG (*p* < 0.05); ^d^P, compared with the THSG + LPS, (*p* < 0.05).

### 3.3 Levels of inflammatory cytokines in HCECs

The expression levels of inflammatory cytokines IL-1β and IL-8 in HCECs differed significantly among the groups (IL-1β: *F* = 4,231.95, *p* < 0.001; IL-8: *F* = 366.66, *p* < 0.001). The baseline levels of IL-1β and IL-8 in the CG were (23.59 ± 1.17 pg/mL) and (55.12 ± 2.15 pg/mL), respectively. Under hyper-osmotic stress, both IL-1β (102.88 ± 4.49 pg/mL) and IL-8 (236.15 ± 64.95 pg/mL) levels in the MG were significantly higher than those in the CG (both *p* < 0.05). With additional LPS stimulation, the inflammatory response in the MG + LPS group was further enhanced, with IL-1β (128.60 ± 5.35 pg/mL) and IL-8 (285.10 ± 9.37 pg/mL) levels being significantly higher than those in the MG (both *p* < 0.05). In the THSG group, IL-1β (56.19 ± 2.51 pg/mL) and IL-8 (148.51 ± 7.80 pg/mL) levels were significantly lower than those in both the MG and MG + LPS (all *p* < 0.05), yet remained significantly higher than those in the CG (*p* < 0.05). The THSG + LPS showed IL-1β (73.20 ± 4.35 pg/mL) and IL-8 (190.12 ± 10.12 pg/mL) levels that were significantly higher than those in the THSG (both *p* < 0.05), but significantly lower than those in both the MG and MG + LPS (both *p* < 0.05). Notably, the TOG group exhibited IL-1β (22.10 ± 1.51 pg/mL) and IL-8 (49.80 ± 2.38 pg/mL) levels that showed no significant difference from the CG and were significantly lower than those in the MG, MG + LPS, THSG, and THSG + LPS (all *p* < 0.05). Details are presented in Table 3 and Figures 2B,C.

**Table 3:** Levels of inflammatory cytokines in HCECs.

Groups	IL-1β (pg/mL)	IL-8 (pg/mL)
CG	23.59 ± 1.17	55.12 ± 2.15
MG	102.88 ± 4.49^bcd^	236.15 ± 64.95^bcd^
MG + LPS	128.60 ± 5.35^acd^	285.10 ± 9.37^acd^
THSG	56.19 ± 2.51^abd^	148.51 ± 7.80^abd^
THSG + LPS	73.20 ± 4.35^abc^	190.12 ± 10.12^abc^
TOG	22.10 ± 1.51	49.80 ± 2.38
*F*	4,231.95	366.66
*p*	<0.001	<0.001

^a^P, compared with the MG (*p* < 0.05); ^b^P, compared with the MG + LPS (*p* < 0.05); ^c^P, compared with the THSG (*p* < 0.05); ^d^P, compared with the THSG + LPS, (*p* < 0.05).

### 3.4 Immunofluorescence of NF-κB p65 nuclear translocation

**Figure 3:**
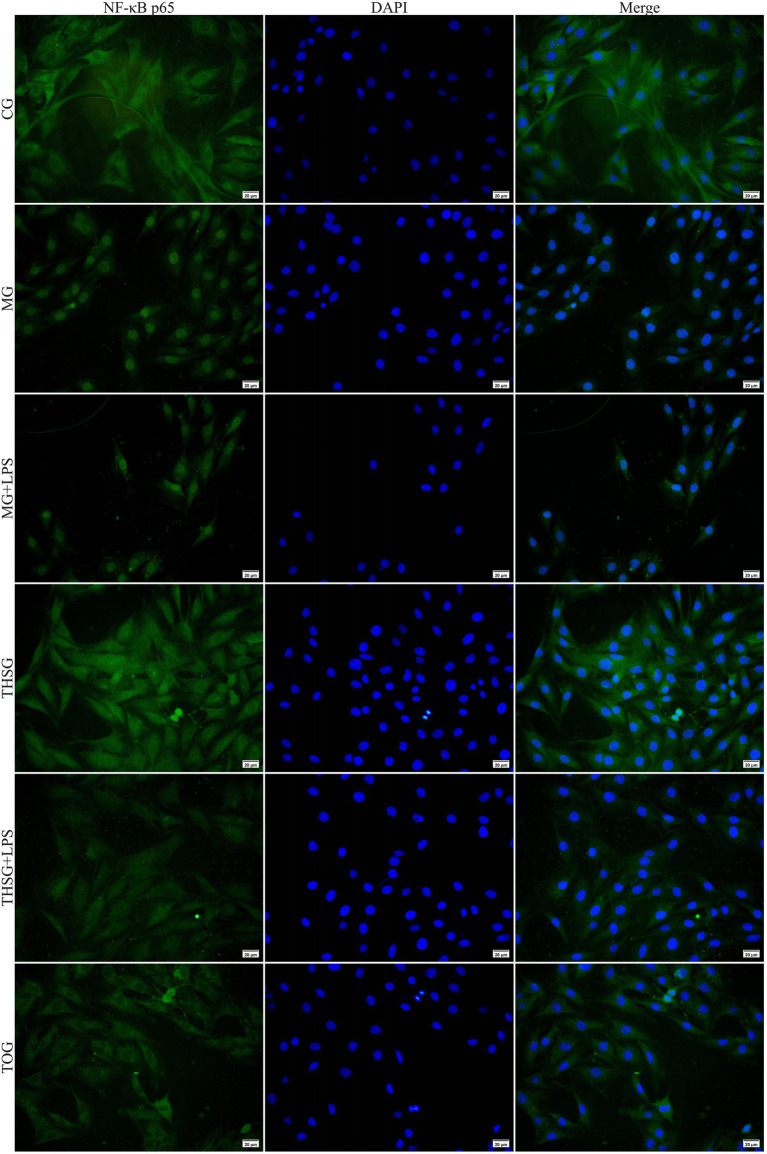
Immunofluorescence analysis of NF-κB p65 nuclear translocation in HCECs under different treatments. Cells were treated for 12 h, stained with an NF-κB p65-specific antibody (green) and the nuclear dye DAPI (blue), and observed under a fluorescence microscope. Representative fields of view are shown. In the control group (CG), p65 signal was predominantly cytoplasmic. Hyperosmotic stress (MG) and hyperosmotic stress plus LPS (MG + LPS) significantly induced nuclear accumulation of p65 protein (green and blue co-localization, appearing as a yellow/white signal). Triptolide intervention (THSG, THSG + LPS) markedly inhibited this nuclear translocation process. Scale bar = 20 μm.

Immunofluorescence staining revealed distinct patterns of NF-κB p65 subcellular localization among the experimental groups (Figure 3). In the CG, NF-κB p65 was predominantly cytoplasmic, with diffuse green fluorescence throughout the cytosol. At the same time, the nuclei (stained blue with DAPI) showed minimal or absent green fluorescence, indicating the quiescent state of NF-κB signaling in normal HCECs. Conversely, exposure to hyperosmotic stress in the MG resulted in prominent nuclear translocation of NF-κB p65, as evidenced by intense green fluorescence signal co-localizing with DAPI-stained nuclei (appearing cyan/white in merged images), indicating robust activation of the NF-κB pathway. This nuclear translocation was further exacerbated in the MG + LPS condition, in which the majority of cells showed substantial nuclear accumulation of p65, with reduced cytoplasmic staining, suggesting synergistic activation of TLR4/NF-κB signaling by hyperosmolarity and LPS stimulation. Pretreatment with triptolide in the THSG markedly attenuated hyperosmotic stress-induced NF-κB p65 nuclear translocation. The green fluorescence signal remained primarily cytoplasmic, with significantly reduced nuclear co-localization compared to the MG, though marginally higher than the CG, indicating that triptolide effectively inhibited p65 nuclear entry. Similarly, in the THSG + LPS, triptolide intervention partially suppressed the LPS-enhanced nuclear translocation observed in MG + LPS, with NF-κB p65 predominantly retained in the cytoplasm; however, the inhibitory effect was less pronounced compared to the THSG group without LPS stimulation. Notably, the TOG exhibited a subcellular distribution pattern comparable to the CG, with NF-κB p65 exclusively localized in the cytoplasm and no detectable nuclear accumulation, confirming that triptolide alone does not induce aberrant NF-κB activation in normal HCECs.

### 3.5 Relative mRNA expression levels of NLRP3 and NF-κB in HCECs

The relative mRNA expression levels of NLRP3 and NF-κB in HCECs showed highly significant differences among all groups (NLRP3: *F* = 85.020, *p* < 0.001; NF-κB: *F* = 113.06, *p* < 0.001). The expression level in the CG was set as 1.00 ± 0.00. Compared with the CG, the hyperosmotic stress MG exhibited significant upregulation in both NLRP3 mRNA (2.33 ± 0.27) and NF-κB mRNA (2.00 ± 0.11) expression (*p* < 0.05). Additional LPS stimulation in the MG + LPS led to a further increase, with NLRP3 mRNA (2.88 ± 0.40) and NF-κB mRNA (2.34 ± 0.25) levels being significantly higher than those in the MG (*p* < 0.05). THSG intervention effectively suppressed this increase, as its NLRP3 mRNA (1.69 ± 0.27) and NF-κB mRNA (1.43 ± 0.15) levels were significantly lower than those in both the MG and MG + LPS (*p* < 0.05). However, they remained significantly higher than in the CG (*p* < 0.05). The expression levels in the THSG + LPS (NLRP3 mRNA: 2.17 ± 0.33; NF-κB mRNA: 1.68 ± 0.12) were significantly higher than those in the THSG (*p* < 0.05) but significantly lower than those in both the MG and MG + LPS (*p* < 0.05). The expression levels in the TOG (NLRP3 mRNA: 0.94 ± 0.09; NF-κB mRNA: 1.06 ± 0.07) showed no significant difference from the CG and were significantly lower than those in all other experimental groups except the CG (*p* < 0.05). Details are presented in Table 4 and Figures 2D,F.

**Table 4:** Relative mRNA expression levels of NLRP3 and NF-κB in HCECs.

Groups	NLRP3 mRNA	NFκB mRNA
CG	1.00 ± 0.00	1.00 ± 0.00
MG	2.33 ± 0.27^bcd^	2.00 ± 0.11^bcd^
MG + LPS	2.88 ± 0.40^acd^	2.34 ± 0.25^acd^
THSG	1.69 ± 0.27^abd^	1.43 ± 0.15^abd^
THSG + LPS	2.17 ± 0.33^abc^	1.68 ± 0.12^abc^
TOG	0.94 ± 0.09	1.06 ± 0.07
*F*	85.020	113.06
*p*	<0.001	<0.001

^a^P, compared with the MG (*p* < 0.05); ^b^P, compared with the MG + LPS (*p* < 0.05); ^c^P, compared with the THSG (*p* < 0.05); ^d^P, compared with the THSG + LPS, (*p* < 0.05).

### 3.6 Relative protein expression levels of NLRP3 and NF-κB in HCECs

The relative protein expression levels of NLRP3 and NF-κB in HCECs also showed highly significant differences among all groups (NF-κB: *F* = 113.060, *p* < 0.001; NLRP3: *F* = 85.020, *p* < 0.001). The protein expression level in the CG (1.00 ± 0.00) served as the baseline. Both NF-κB (1.70 ± 0.27) and NLRP3 (1.82 ± 0.35) protein expression in the MG were significantly higher than those in the CG (*p* < 0.05). The protein expression levels in the MG + LPS (NF-κB: 1.81 ± 0.18; NLRP3: 1.96 ± 0.26) showed no significant difference from the MG but were significantly higher than those in the CG (*p* < 0.05). THSG intervention significantly reduced protein expression. It’s NF-κB (1.41 ± 0.13) and NLRP3 (1.40 ± 0.16) levels were significantly lower than those in both the MG and MG + LPS (*p* < 0.05), though they remained significantly higher than in the CG (*p* < 0.05). The protein expression in the THSG + LPS (NF-κB: 1.58 ± 0.26; NLRP3: 1.56 ± 0.30) was significantly higher than that in the THSG (*p* < 0.05), while its NLRP3 expression was significantly lower than that in the MG + LPS (*p* < 0.05). The protein expression in the TOG (NF-κB: 0.93 ± 0.15; NLRP3: 1.09 ± 0.11) was restored to a level showing no significant difference from the CG and was significantly lower than that in all other experimental groups except the CG (*p* < 0.05). Details are presented in Table 5 and Figures 2E,G,K,L.

**Table 5:** Relative protein expression levels of NLRP3 and NF-κB in HCECs.

Groups	NFκB	NLRP3
CG	1.00 ± 0.00	1.00 ± 0.00
MG	1.70 ± 0.27^bcd^	1.82 ± 0.35^cd^
MG + LPS	1.81 ± 0.18^acd^	1.96 ± 0.26^cd^
THSG	1.41 ± 0.13^abd^	1.40 ± 0.16^ac^
THSG + LPS	1.58 ± 0.26^abc^	1.56 ± 0.30^abc^
TOG	0.93 ± 0.15	1.09 ± 0.11
*F*	113.060	85.020
*p*	<0.001	<0.001

^a^P, compared with the MG (*p* < 0.05); ^b^P, compared with the MG + LPS (*p* < 0.05); ^c^P, compared with the THSG (*p* < 0.05); ^d^P, compared with the THSG + LPS, (*p* < 0.05).

### 3.7 Levels of pyroptosis-related indicators (GSDMD-N, LDH release, and pyroptosis rate) in HCECs

The pyroptosis-related indicators in HCECs—GSDMD-N protein expression, LDH release, and pyroptosis rate—showed highly significant differences among all groups (GSDMD-N: *F* = 920.155, *p* < 0.001; LDH release: *F* = 6,973.693, *p* < 0.001; Pyroptosis rate: *F* = 1,187.952, *p* < 0.001). Details are presented in Table 6; Figures 2H,I,J,M.

**Table 6:** Levels of pyroptosis-related indicators (GSDMD-N, LDH release, and pyroptosis rate) in HCECs.

Groups	GSDMD-N	LDH release from HCECs (U/L)	Pyroptosis rate of HCECs (%)
CG	1.00 ± 0.00	33.92 ± 1.71	5.40 ± 0.81
MG	5.33 ± 0.57^bcd^	286.45 ± 14.17^bcd^	40.28 ± 3.74^bcd^
MG + LPS	7.51 ± 0.74^acd^	375.58 ± 10.41^acd^	46.12 ± 3.16^acd^
THSG	2.37 ± 0.37^abd^	96.30 ± 2.65^abd^	17.01 ± 2.33^abd^
THSG + LPS	4.91 ± 0.46^abc^	218.95 ± 6.04^abc^	25.88 ± 4.14^abc^
TOG	1.29 ± 0.16	28.35 ± 2.42	2.78 ± 1.06
*F*	920.155	6,973.693	1,187.952
*p*	<0.001	<0.001	<0.001

^a^P, compared with the MG (*p* < 0.05); ^b^P, compared with the MG + LPS (*p* < 0.05); ^c^P, compared with the THSG (*p* < 0.05); ^d^P, compared with the THSG + LPS, (*p* < 0.05).

All indicators in the CG remained at baseline levels: the relative expression of GSDMD-N was 1.00 ± 0.00, LDH release was (33.92 ± 1.71) U/L, and the cellular pyroptosis rate was (5.40 ± 0.81)%.

Under hyperosmotic stress, pyroptosis was significantly activated in the MG. Its GSDMD-N expression (5.33 ± 0.57), LDH release (286.45 ± 14.17 U/L), and pyroptosis rate (40.28 ± 3.74%) were all significantly higher than those in the CG (*p* < 0.05). When the MG was co-stimulated with LPS (MG + LPS), all indicators were further exacerbated: GSDMD-N (7.51 ± 0.74), LDH release (375.58 ± 10.41 U/L), and pyroptosis rate (46.12 ± 3.16%) were all significantly higher than those in the MG (*p* < 0.05).

THSG intervention demonstrated significant anti-pyroptotic effects. All indicators in the THSG (GSDMD-N: 2.37 ± 0.37; LDH: 96.30 ± 2.65 U/L; Pyroptosis rate: 17.01 ± 2.33%) were significantly lower than those in both the MG and MG + LPS (*p* < 0.05), though they remained significantly higher than in the CG (*p* < 0.05). However, adding LPS to THSG (THSG + LPS) partially reversed this protective effect. The levels in this group (GSDMD-N: 4.91 ± 0.46; LDH: 218.95 ± 6.04 U/L; Pyroptosis rate: 25.88 ± 4.14%) were significantly higher than those in the THSG (*p* < 0.05) but significantly lower than those in both the MG and MG + LPS (*p* < 0.05).

**Figure 4:**
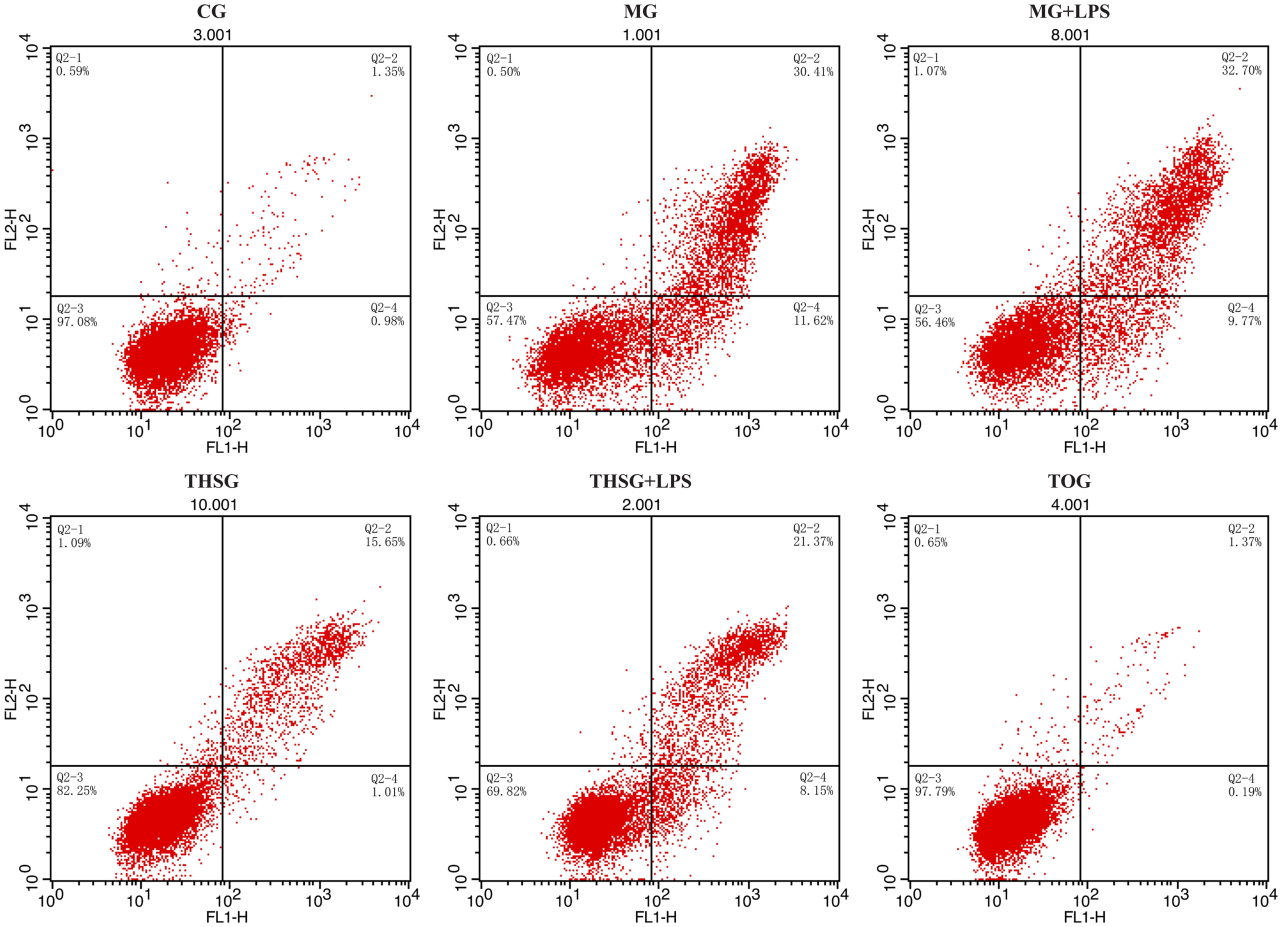
The pyroptosis rate of HCECs (%). The pyroptosis rate of HCECs in each group was quantitatively detected by flow cytometry (Annexin V-FITC/PI double staining). Control group (CG); the significant pathological changes such as cell shrinkage and rounding induced by hyperosmotic stress (MG) and hyperosmotic stress plus LPS (MG + LPS); the amelioration of these hyperosmosis-induced changes by triptolide intervention (THSG); the partial attenuation of this protective effect when triptolide was co-administered with LPS under hyperosmotic stress (THSG + LPS); and the similar morphology to CG in the triptolide-only group (TOG).

Most notably, the TOG exhibited nearly complete protective efficacy against all pyroptosis indicators. Its GSDMD-N expression (1.29 ± 0.16), LDH release (28.35 ± 2.42 U/L), and pyroptosis rate (2.78 ± 1.06%) did not differ significantly from those in the CG. It was simultaneously significantly lower than those in all other experimental groups—MG, MG + LPS, THSG, and THSG + LPS (*p* < 0.05) (Figure 4).

## 4 Discussion

This study reveals the molecular mechanism by which triptolide protects HCECs against hyperosmotic stress-induced damage by modulating the TLR4/NF-κB/NLRP3 signaling axis. The results indicate that a 500 mOsm hyperosmotic environment significantly inhibits HCECs proliferation and induces pyroptosis, accompanied by a marked increase in the levels of inflammatory cytokines IL-1β and IL-8, as well as activation of the TLR4/NF-κB signaling pathway and the NLRP3 inflammasome at both gene and protein levels, manifested as enhanced nuclear translocation of the NF-κB p65 subunit. Notably, pretreatment with 30 nM triptolide significantly reversed these pathological alterations: it effectively promoted cell proliferation, reduced the pyroptosis rate, inhibited the release of inflammatory factors, downregulated the expression of NF-κB and NLRP3, and blocked the nuclear translocation of NF-κB p65. Furthermore, Triptolide alone showed no cytotoxicity toward normal HCECs and even exhibited a certain anti-apoptotic tendency, suggesting favorable biosafety. In summary, this study proposes that triptolide likely alleviates hyperosmotic stress-induced pyroptosis and inflammatory injury in HCECs by inhibiting TLR4/NF-κB-mediated activation of the downstream NLRP3 inflammasome, thereby providing experimental evidence for the potential use of this compound to prevent and treat such cellular damage.

Hyper-osmotic stress is a central pathological event in dry eye disease, inducing inflammatory molecules such as MMP-9 and IL-1 (via MAPK pathways, including JNKs and ERK), leading to cell death (1, 15). Triptolide treatment markedly reduced IL-1β and IL-8 levels in the present study. This is in line with previous reports that triptolide blocked NF-κB-dependent expression of IL-8 and monocyte chemoattractant protein-1 (MCP-1) (16), providing evidence for its broad bioactivity in hyperosmolar-induced pro-inflammatory pathways. Because mature IL-1β is an immediate downstream product of NLRP3 inflammasome activation, its decreased secretion directly demonstrates the inhibitory effect of Triptolide at NLRP3 inflammasome (17). Furthermore, triptolide’s remarkable inhibition of pyrolysis implied that its protective effect could involve not only the blockade of other apoptotic pathways but also increased cell survival by blocking pyroptosis, possibly through targeting caspase-1/GSDMD. This is supported by previous reports showing that NLRP3 inflammasome activation triggers pyroptotic cell death via caspase-1-mediated cleavage of GSDMD, leading to cell membrane rupture (18). Our preliminary data on lactate dehydrogenase (LDH) release and cell morphological changes further corroborate triptolide’s inhibitory effect on pyroptosis.

The TLR4 acts as a pivotal trigger activated by hyper-osmotic stress. Its binding activates a signaling cascade that leads to the nuclear translocation of NF-κB, which then increases NLRP3 transcription (11). Western blotting showed that triptolide could inhibit the phosphorylation of IκBα (p-IκBα) andblock NF-κB p65 nuclear translocation. This result is consistent with previous studies showing that triptolide inhibits NF-κB activity by up-regulating IκBα in lung inflammation models (19). qPCR results also showed that NLRP3 mRNA expression decreased following Triptolide treatment, supporting the TLR4/NF-κB pathway as an essential upstream mediator of NLRP3 transcription. Unlike the targeted anti-inflammatory action of JNK inhibitors or glucocorticoids alone, triptolide provides broader-spectrum coverage by simultaneously inhibiting not only the upstream TLR4/NF-κB but also the downstream NLRP3 inflammasome, thereby promoting synergistic anti-inflammatory and cytoprotective activity (11–14).

Additionally, this study further validated the mechanism of Triptolide by using LPS as a TLR4 agonist. The experimental results showed that under hyperosmotic conditions, LPS stimulation (MG + LPS) significantly aggravated pathological processes, including NF-κB p65 nuclear translocation, up-regulation of NLRP3 expression, and pyroptosis, indicating effective activation of TLR4 signaling. Triptolide intervention (THSG + LPS) markedly suppressed these LPS-enhanced effects; although its protective effect was somewhat attenuated compared to the THSG without LPS, it remained significantly superior to the untreated groups. These findings further support the notion that Triptolide exerts its protective effect by inhibiting the TLR4/NF-κB pathway, demonstrating considerable inhibitory efficacy even when TLR4 signaling is potentiated by an exogenous agonist such as LPS, thereby providing more direct experimental evidence for its targeted intervention in TLR4-dependent inflammatory responses.

The hyper-osmotic environment may also trigger inflammasome activation via reactive oxygen species (ROS) within the cell. This is in line with findings in macrophages, where hyperosmolar-driven NLRP3 activation generates a powerful inflammatory amplifier (20). In the present study, triptolide significantly reduced the protein levels of key pyroptosis components—NLRP3 and GSDMD-N. These results robustly indicate that triptolide blocks the canonical NLRP3-ASC-caspase-1-GSDMD pyroptosis pathway. These results powerfully demonstrate that triptolide suppresses the canonicalNLRP3-ASC-caspase-1-GSDMD pyroptosis pathway. A similar inhibitory effect was reported with calcitriol in a dry eye model, where calcitriol reduced pyroptosis by suppressing the NLRP3-ASC-caspase-1-GSDMD axis (21), further confirming the critical role for this pathway in hyperosmolar-induced corneal epithelial injury. It is noteworthy that the function of NLRP3 in corneal epithelial cells extends beyond inflammation to include the direct regulation of cell death decisions. Therefore, the dual inhibitory action of triptolide—targeting both the upstream TLR4/NF-κB signal and the downstream NLRP3 inflammasome assembly—effectively disrupts the inflammatory amplification cycle, leading to a significant reduction in both IL-1β maturation/release and pyroptotic cell death.

Notably, results from the TOG confirmed that 30 nM Triptolide had no adverse effects on the proliferation, inflammatory marker expression, or overall health of normal HCECs. Intriguingly, it even slightly reduced the cells’ death rate, indicating excellent cellular tolerance and a potential pro-survival effect at this concentration. This finding is supported by previous work showing no cytotoxicity in corneal fibroblasts within the 1–30 nM range and concurrent inhibition of NF-κB activation (16), which provided the rationale for the dose selected in our study. Notably, compared to glucocorticoids, which are associated with side effects such as elevated intraocular pressure and cataract formation upon long-term ocular application (11), triptolide, a naturally derived diterpenoid, demonstrates a wider therapeutic window and significant potential for clinical translation.

In this study, immunofluorescence assays revealed that hyper-osmotic stress (500 mOsm) significantly promoted the nuclear translocation of NF-κB p65, while pretreatment with 30 nM triptolide markedly suppressed this process. These findings suggest that hyperosmotic stress facilitates the translocation of p65 from the cytoplasm to the nucleus by activating the TLR4/NF-κB pathway, thereby initiating transcription of the NLRP3 inflammasome and downstream pyroptosis-related factors, such as IL-1β and IL-8. By blocking the nuclear translocation of p65, triptolide effectively disrupts the NF-κB-dependent amplification loop of inflammatory signaling, thereby alleviating pyroptosis and injury in corneal epithelial cells. Previous studies have demonstrated that in corneal fibroblasts, triptolide inhibits IL-1β- or poly(I: C)-induced NF-κB activation and reduces the nuclear accumulation of p65 (22). Similarly, in renal podocytes, triptolide has been shown to suppress the entire NF-κB signaling cascade by inhibiting IκBα phosphorylation and p65 nuclear translocation (23). These consistent findings across different experimental models indicate that inhibition of NF-κB nuclear translocation is a core mechanism underlying triptolide’s anti-inflammatory effects.

The protective effect of triptolide observed in the present study is also in line with its known inhibitory function against NLRP3, as shown in models mimicking lung inflammation, neuroinflammation, and kidney injury, where it reduces NLRP3 expression either by preventing TLR4/NF-κB activation or by directly stabilizing IκBα (11–19). We systematically validate, for the first time using a dry eye-related hyper-osmotic corneal epithelial cell model, a mechanism that is thereby rendered expansible to ocular surface pathology. Although studies of hyper-osmotic corneal injuries now mainly emphasize the MAPK pathway or a single inflammatory mediator, they have rarely involved research on the whole TLR4/NF-κB-NLRP3regulatory axis. To the best of our knowledge, we are the first to discover that triptolide can simultaneously impair the TLR4 inflammasome activation cascade, NF-κB nuclear translocation, and NLRP3 inflammasome complex formation under hyper-osmotic stress, thereby filling a significant void in multi-target therapeutic options for OSDs. By combining our mRNA and protein expression data with functional studies of apoptosis and pyroptosis, we provide a body of evidence that spans transcriptional control to the execution of cell death. This multi-aspect compound intervention strongly supports the pathological “cascade” of hyperosmotic stress→TLR4/NF-κB activation→NLRP3 inflammasome assembly pyroptosis. This concept nicely explains the latest reports on the ROS-NLRP3 axis in dry eye (20).

This study has several main limitations: the model system is relatively simple, relying solely on an *in vitro* monolayer corneal epithelial cell model that does not fully replicate the complex ocular surface microenvironment or *in vivo* pathophysiology, necessitating further validation in animal models; while the use of LPS to activate TLR4 and the observed reversal by triptolide provide strong pharmacological support for TLR4/NF-κB pathway involvement, more direct causal evidence—such as through TLR4-knockout cells—is needed to firmly establish necessity and exclude contributions from parallel pathways; the experimental conditions are relatively fixed, examining only a single concentration (30 nM) and time point (12 h) without defining the optimal therapeutic window or the compound’s concentration–response and time–kinetic relationships; observations of pyroptosis, though supported by downstream indicators (GSDMD-N, LDH), lack direct examination of key upstream execution events like caspase-1 activation and ASC speck formation, limiting mechanistic completeness; translational application faces formulation challenges, as triptolide’s potential poor solubility and corneal permeability were not addressed, and ocular delivery systems remain unexplored; finally, the pathological complexity is incompletely captured, as the focus on epithelial cells alone without immune cell co-culture fails to model the inflammatory interaction network among ocular surface cell types under hyperosmotic stress. Future studies should address these gaps through *in vivo* validation, mechanistic confirmation using genetic and pharmacological tools, systematic parameter screening, novel ocular formulation development, and the use of more complex cellular models.

## 5 Summary

Based on *in vitro* experiments, this study suggests that triptolide may alleviate hyperosmotic stress-induced pyroptosis and inflammation in human corneal epithelial cells by inhibiting the TLR4/NF-κB signaling pathway and the downstream activation of the NLRP3 inflammasome. The results indicate that pretreatment with 30 nM triptolide improved cell proliferation, reduced pyroptosis-related indicators (GSDMD-N, LDH release) and levels of inflammatory cytokines (IL-1β, IL-8), and suppressed nuclear translocation of NF-κB p65 and NLRP3 expression. Under normal culture conditions, this concentration did not exhibit apparent cytotoxicity, indicating favorable biocompatibility within the tested range. It should be noted that the above conclusions are derived from a cell-based model, and there in vivo relevance and clinical translatability require further investigation. Future studies should include validation in animal models and exploration of appropriate delivery routes and formulations. This work provides preliminary experimental evidence for further investigation into triptolide’s role in dry eye and related ocular surface disorders.

## Data Availability

The raw data supporting the conclusions of this article will be made available by the authors without undue reservation.
